# Supplementary material for: A fault-intrusion-tolerant system and deadline-aware algorithm for scheduling scientific workflow in the cloud
Source: PeerJ Comput Sci. 2021 Nov 2;7:e747. doi: 10.7717/peerj-cs.747 (PMC8576554; doi:10.7717/peerj-cs.747)
Supplement: Supplemental Information 6 [file peerj-cs-07-747-s006.zip › Myproject1.1/docs/index-files/index-5.html]

E-Index


JavaScript is disabled on your browser.


- Overview
- Package
- Class
- Use
- Tree
- Deprecated
- Index
- Help

- Prev Letter
- Next Letter

- Frames
- No Frames

- All Classes

A B C D E F G H I J L M N O P Q R S T U V W Z 


## E

EDGE\_LEVEL - Static variable in class org.cloudbus.cloudsim.network.datacenter.NetworkConstants


EdgeSwitch - Class in org.cloudbus.cloudsim.network.datacenter
:   This class allows to simulate Edge switch for Datacenter network.

EdgeSwitch(String, int, NetworkDatacenter) - Constructor for class org.cloudbus.cloudsim.network.datacenter.EdgeSwitch
:   Constructor for Edge Switch We have to specify switches that are connected to its downlink
    and uplink ports, and corresponding bandwidths.

EdgeSwitchPort - Static variable in class org.cloudbus.cloudsim.network.datacenter.NetworkConstants


enable() - Static method in class org.cloudbus.cloudsim.Log
:   Enables the output.

ENABLE\_OUTPUT - Static variable in class org.cloudbus.cloudsim.examples.power.Constants


end(String) - Static method in class org.cloudbus.cloudsim.util.ExecutionTimeMeasurer
:   End.

END\_OF\_SIMULATION - Static variable in class org.cloudbus.cloudsim.core.CloudSimTags
:   Denotes the end of simulation

endWaitingTime() - Method in class org.cloudbus.cloudsim.core.SimEvent
:   Get the simulation time that this event was removed from the queue for service.

engine - Variable in class org.workflowsim.ClusteringEngine
:   The clustering engine to use

ENULL - Static variable in class org.cloudbus.cloudsim.core.SimEvent


eventTime() - Method in class org.cloudbus.cloudsim.core.SimEvent
:   Get the simulation time that this event was scheduled.

EXECUTION - Static variable in class org.cloudbus.cloudsim.network.datacenter.NetworkConstants


ExecutionTimeMeasurer - Class in org.cloudbus.cloudsim.util
:   The class for measuring the execution time.

ExecutionTimeMeasurer() - Constructor for class org.cloudbus.cloudsim.util.ExecutionTimeMeasurer


exeTime - Variable in class org.cloudbus.cloudsim.network.datacenter.AppCloudlet


exetime - Variable in class org.cloudbus.cloudsim.network.datacenter.NetworkCloudlet


EXPERIMENT - Static variable in class org.cloudbus.cloudsim.core.CloudSimTags
:   Sends an Experiment object between UserEntity and Broker entity

ExponentialDistr - Class in org.cloudbus.cloudsim.distributions
:   An exponential number generator.

ExponentialDistr(long, double) - Constructor for class org.cloudbus.cloudsim.distributions.ExponentialDistr
:   Creates a new exponential number generator.

ExponentialDistr(double) - Constructor for class org.cloudbus.cloudsim.distributions.ExponentialDistr
:   Creates a new exponential number generator.

extractHostListFromMigrationMap(List<Map<String, Object>>) - Method in class org.cloudbus.cloudsim.power.PowerVmAllocationPolicyMigrationAbstract
:   Extract host list from migration map.

A B C D E F G H I J L M N O P Q R S T U V W Z

- Overview
- Package
- Class
- Use
- Tree
- Deprecated
- Index
- Help

- Prev Letter
- Next Letter

- Frames
- No Frames

- All Classes
